# Supplementary material for: Glycerol-Mediated Facile Synthesis of Colored Titania Nanoparticles for Visible Light Photodegradation of Phenolic Compounds
Source: Nanomaterials (Basel). 2019 Nov 8;9(11):1586. doi: 10.3390/nano9111586 (PMC6915729; doi:10.3390/nano9111586)
Supplement: Supplementary file 1 [file nanomaterials-09-01586-s001.pdf]

Supplementary Materials

Glycerol-Mediated Facile Synthesis of Colored Titania Nanoparticles for Visible Light Photodegradation of Phenolic Compounds

Rab Nawaz [1,*], Chong Fai Kait [1,*], Ho Yeek Chia [2], Mohamed Hasnain Isa [3] and Lim Wen Huei [4]

1 Fundamental and Applied Sciences Department, Universiti Teknologi PETRONAS, Seri Iskandar 32610, Perak, Malaysia
2 Civil and Environmental Engineering Department, Universiti Teknologi PETRONAS, Seri Iskandar 32610, Perak, Malaysia; yeekchia.ho@utp.edu.my
3 Civil Engineering programme, Faculty of Engineering, Universiti Teknologi Brunei, Tungku Highway, Gadong BE1410, Brunei Darussalam; hasnain_isa@yahoo.co.uk
4 Advanced Oleochemical Technology Division (AOTD), Malaysian Palm Oil Board (MPOB), Bandar Baru Bangi 43000, Kajang, Selangor, Malaysia; limwen@mpob.gov.my
* Correspondence: rab_17000005@utp.edu.my (R.N.); chongfaikait@utp.edu.my (C.F.K.); Tel.: +60-143056299 (R.N.); +60-182104182 (C.F.K.)

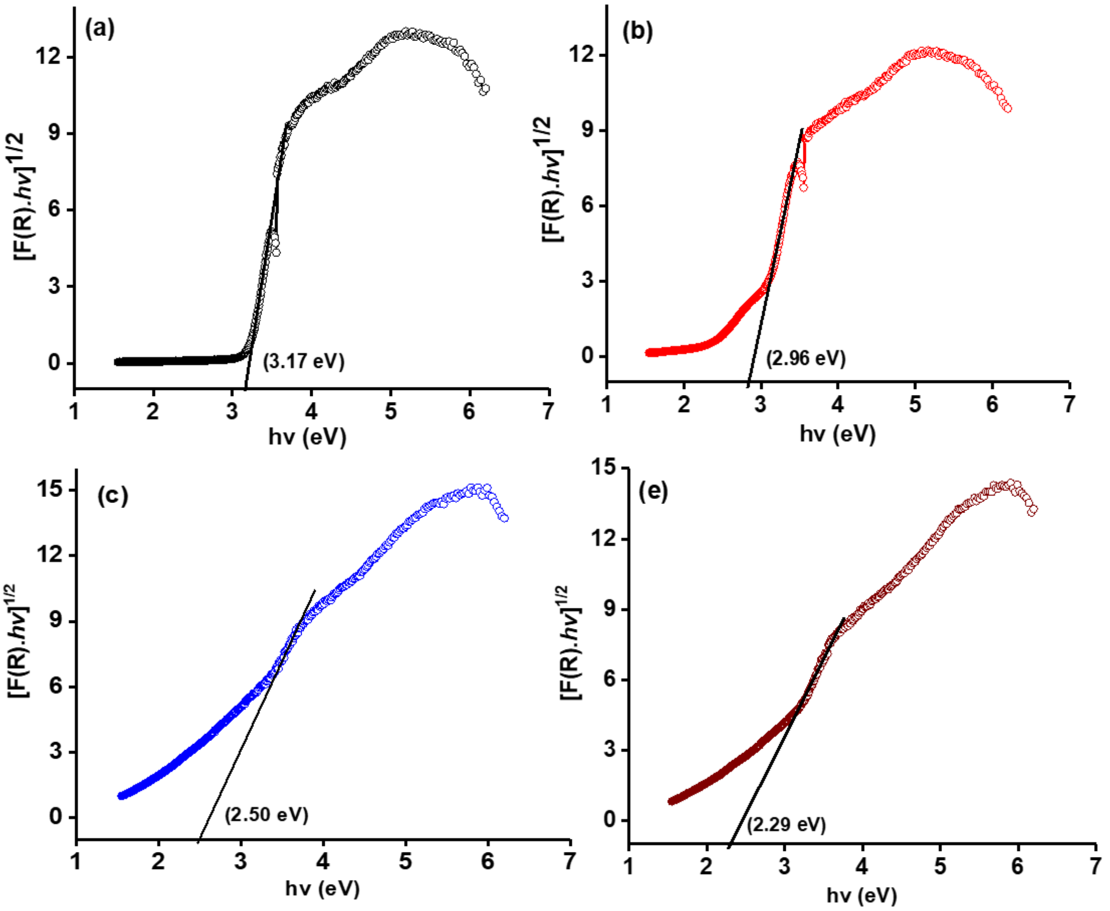

Figure S1. Tauc's plot of the band gaps of the colored TiO_2 NPs prepared at various glycerol concentration and calcination at 300°C: (**a**) T1, (**b**) T2, (**c**) T3, and (**d**) T4.

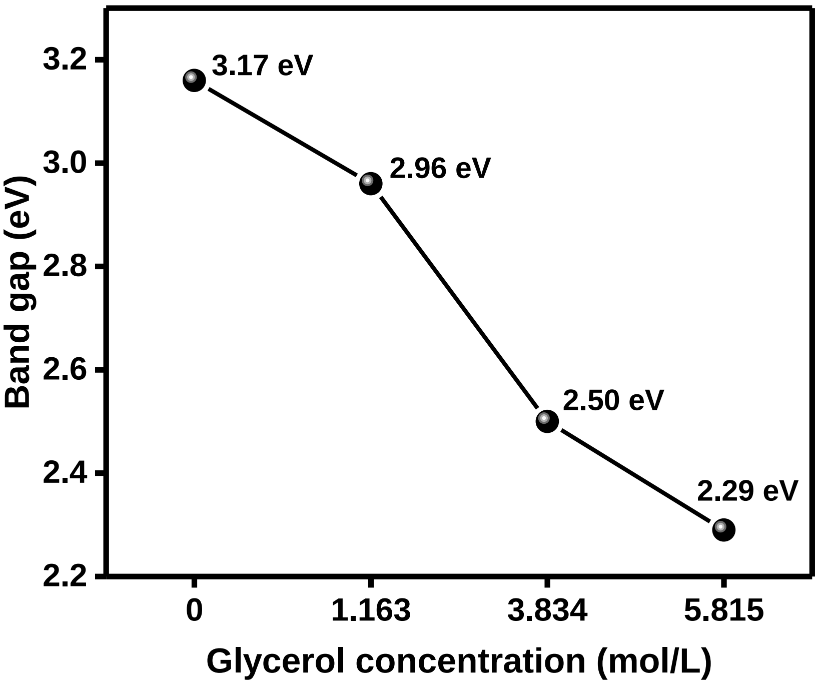

Figure S2. Band gap energy of colored TiO_2 NPs as a function of glycerol concentration.

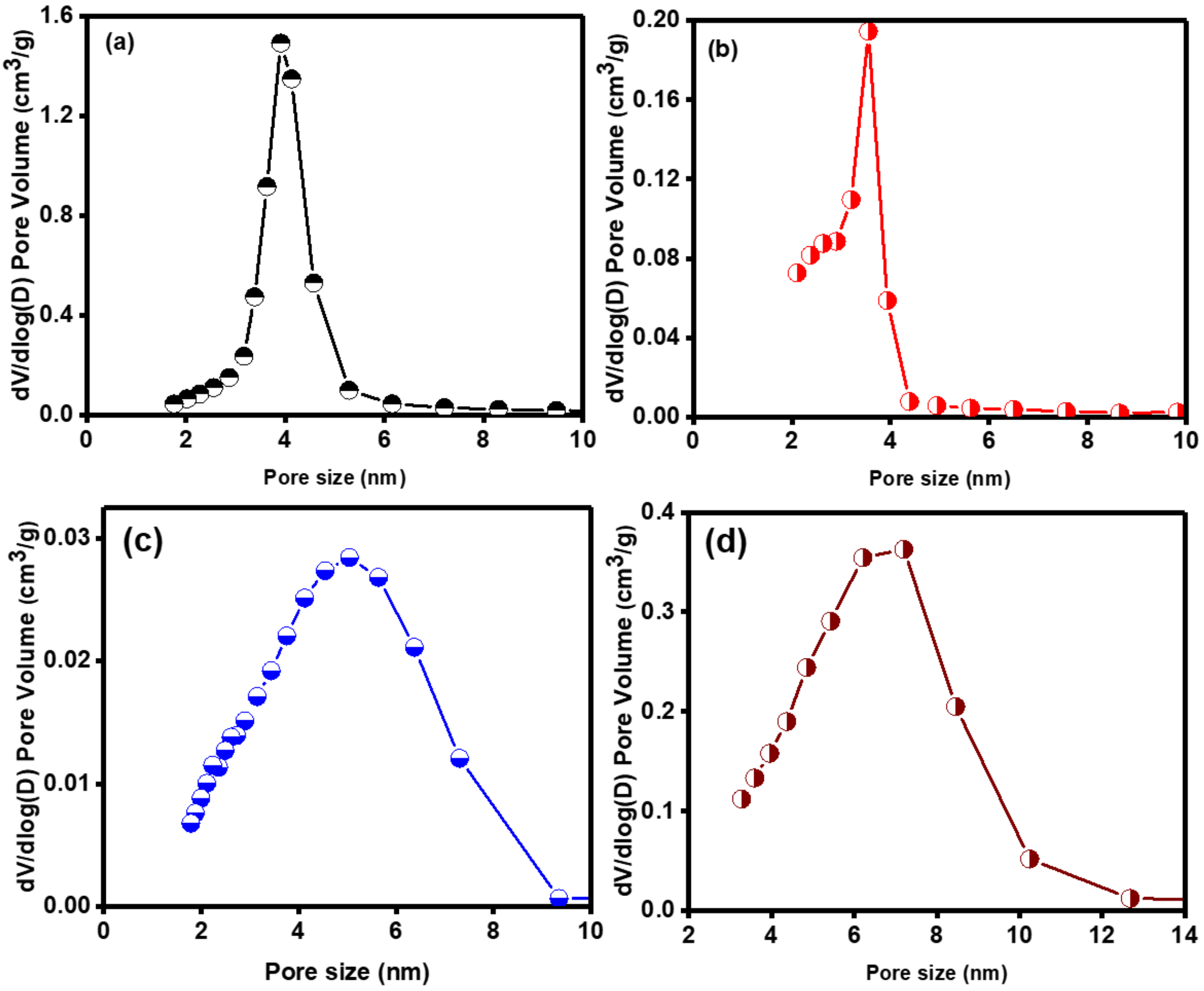

Figure S3. Pore size distribution of colored TiO_2 NPs prepared at various glycerol concentration and calcination at 300°C: (**a**) T1, (**b**) T2, (**c**) T3, and (**d**) T4.

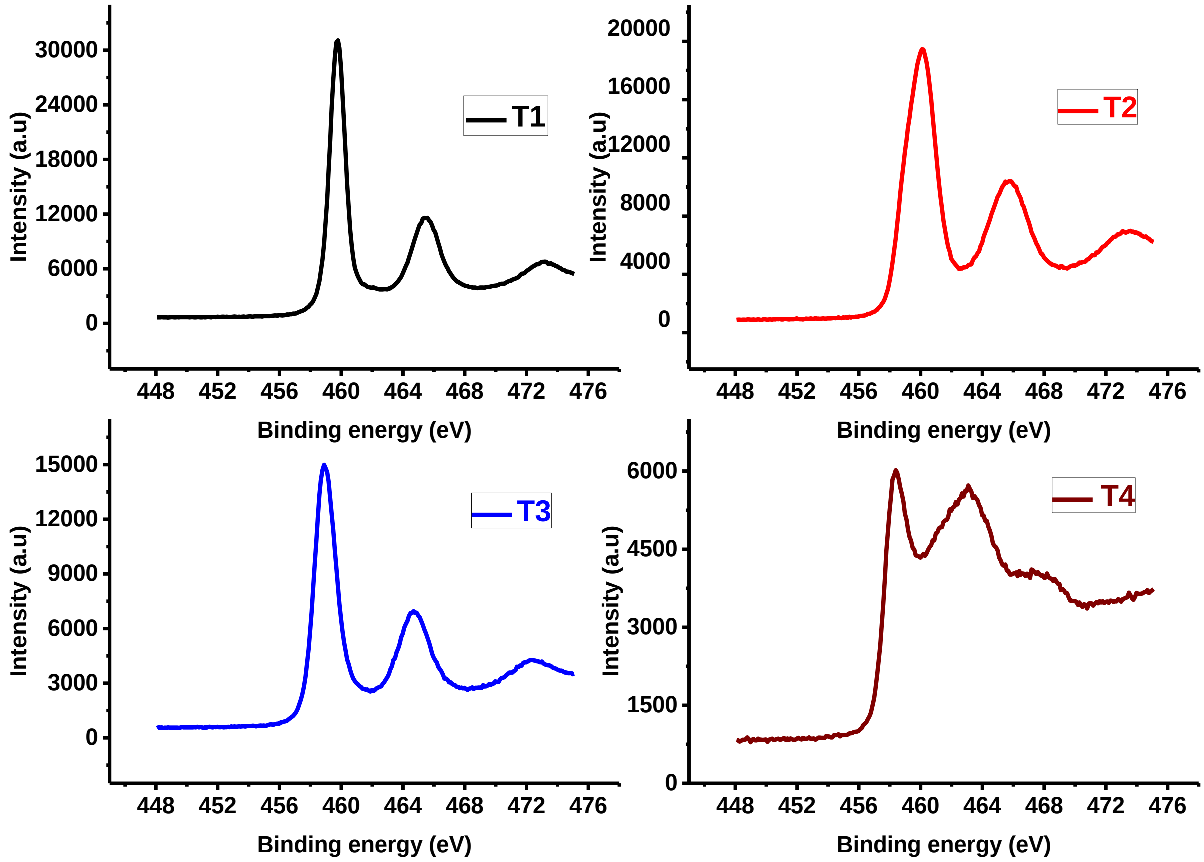

Figure S4. High resolution XPS spectra of Ti2p of the colored TiO_2 NPs prepared at different glycerol concentration 0 mol/L (T1), 1.163 mol/L (T2), 3.834 mol/L (T3) and 5.815 mol/L (T4).

Table S1. XPS fitting parameters the Ti2p$_{3/2}$ and Ti2p$_{1/2}$ and O1s peaks for the colored TiO_2 (Exp.) and values reported in the literature, O_L represents lattice oxygen and O_s sub oxide oxygen.

**Sample**	**Glycerol concentration (mol/L)**	**Ti^{4+}**		**Ti^{3+}**		**O1s**		Ref.
		**Ti2p$_{3/2}$**	**Ti2p$_{1/2}$**	**Ti2p$_{3/2}$**	**Ti2p$_{1/2}$**	**O_L**	**O_s**	
T1	0	459.08	464.53	---	---	530.95	---	This work
T2	1.163	460.07	466.30	458.80	---	530.20	531.70	
T3	3.834	458.95	464.65	---	460.07	530.28	532.48	
T4	5.815	460.00	467.02	458.32	---	529.85	531.70	
Anatase TiO_2	---	458.60	464.40	---	460.40	529.90	531.30	[1]
	---	459.65	---	---	---	530.87	---	[2]
	---	459.00	---	---	---	530.40	---	[3]

Table S2. Difference between binding energies and line separation of the colored TiO_2.

**Sample**	**BE Ti2p$_{1/2}$ – BE Ti2p$_{3/2}$**	**ΔBE/eV**	**BE O1s – BE Ti2p$_{3/2}$**	**ΔBE/eV**	**Ref.**
T1	464.53 – 459.08	5.45	530.95 – 459.08	71.87	This work
T2	466.30 – 460.07	6.23	531.70 – 458.80	72.90	
T3	464.65 – 458.95	5.70	530.28 – 458.95	71.33	
T4	467.02 – 460.00	7.02	531.70 – 458.32	73.38	
Black TiO_2	464.36 – 458.60	5.76	530.54 – 459.25	71.29	[4]

Reference

1. B. Bharti, S. Kumar, H.-N. Lee, and R. Kumar, Formation of oxygen vacancies and Ti^{3+} state in TiO_2 thin film and enhanced optical properties by air plasma treatment, *Scientific Reports* **2016**, *6*, 32355.
2. R. Shvab, E. Hryha, and L. Nyborg, Surface chemistry of the titanium powder studied by XPS using internal standard reference, *Powder Metallurgy,* **2017,** *60*, 42–48.
3. M. C. Biesinger, L. W. Lau, A. R. Gerson, and R. S. C. Smart, Resolving surface chemical states in XPS analysis of first row transition metals, oxides and hydroxides: Sc, Ti, V, Cu and Zn, *Applied Surface Science,* **2010**, *257*, 887–898.
4. S. Chen, Y. Xiao, Y. Wang, Z. Hu, H. Zhao, and W. Xie, A facile approach to prepare black TiO_2 with oxygen vacancy for enhancing photocatalytic activity, *Nanomaterials,* **2018**, *8*, 245.
